# The Physicochemical and Rheological Properties of Green Banana Flour–Wheat Flour Bread Substitutions

**DOI:** 10.3390/plants14020207

**Published:** 2025-01-13

**Authors:** Yasmeen M. Bashmil, Frank Bekes, Michael Ruderman, Hafiz A. R. Suleria, Rudi Appels, Frank R. Dunshea

**Affiliations:** 1 Department of Food and Nutrition, Faculty of Human Sciences and Design, King Abdulaziz University, Jeddah 21589, Saudi Arabia; ybashmil@student.unimelb.edu.au; 2 Faculty of Science, School of Agriculture, Food and Ecosystem Sciences, The University of Melbourne, Parkville, VIC 3010, Australia; mruderman@student.unimelb.edu.au (M.R.); hafiz.suleria@unimelb.edu.au (H.A.R.S.); fdunshea@unimelb.edu.au (F.R.D.); 3 FBFD Pty Ltd., Sydney, NSW 2151, Australia; firinc47@gmail.com; 4 Faculty of Biological Sciences, University of Leeds, Leeds LS2 9JT, UK

**Keywords:** functional bread, green banana, MixoLab, RS, DF, dough rheology, crumb cell structure, bread color

## Abstract

Functional foods are currently receiving increasing popularity in diet modification. Green bananas contain far more dietary fiber (DF) and resistant starch (RS) than mature bananas. The potential for integrating these vital components into food, such as bread, has expanded. Thus, this study aimed to examine the physicochemical and rheological behavior of wheat flour dough after the addition of varying amounts of Australian, green banana flour (GBF) substitutions (5, 10, 15, 25, and 30%). Using MixoLab 2, we recorded the rheological parameters of the dough that had GBF substitutions. Additionally, the flour color (‘L*’, ‘a*’, and ‘b*’ value) and crumb cell structure analysis were evaluated. Although increasing the amount of GBF replacement generally improved dough quality with all banana cultivars, GBF from Cavendish and Ladyfinger showed a greater improvement than Ducasse. Improved dough mixing stability and increased viscosity, starch gelatinization, and retrogradation were all predicted to contribute to longer bread shelf life. RS content of the enriched bread increased significantly with both Ladyfinger and Ducasse (2.6%), while Ladyfinger bread had the highest DF (9.1%). With increasing GBF, L*, a*, and b* values were changed considerably with a strong linear correlation. A MATLAB analysis indicated substantial variations across samples regarding the small, medium, and total air space counts based on 10% banana flour as a standard level of addition. In conclusion, the processing properties and nutritional value of wheat flour can be enhanced by replacing specific proportions of wheat flour with green banana flour without major detrimental effects on dough processing attributes and thus highlight the possibility of utilizing GBF from different banana varieties for use in fine-tuning composite flour developments.

## 1. Introduction

Bread is a staple food closely related to people’s daily life. Despite the availability of a diverse selection of bread types, white bread remains the most popular choice among consumers because of its sensory qualities despite being classified as a high-glycemic-index food. This classification is a result of its high proportion of rapidly digested starch. Additionally, the removal of bran during the production of white wheat flour results in substantial losses of fiber, vitamins (particularly B vitamins and vitamin E), minerals (such as iron and magnesium), and antioxidants, compared to whole wheat flour [1]. Therefore, there is increased interest in the fortification of bread with a variety of dietary fiber (DF) and functional ingredients to benefit from the bread’s potential as a carrier of health-promoting compounds. In recent years, bran, prebiotics, vitamins, minerals, and other functional constituents have been suggested as alternatives to wheat flour [2].

Banana is a tropical climacteric fruit and is considered one of the most popular fruits in the world and the fourth most significant crop produced globally [3]. Green bananas contain far more flavonoids, DF, and resistant starch (RS) than mature bananas [4,5] as well as a higher antioxidant capacity than certain cereals, herbs, and vegetables due to the presence of phenolics, carotenes, flavonoids, and other phytochemicals [6]. An important characteristic of RS is that it does not break down in the small intestine, and it promotes the proliferation of probiotics in the colon, which may have an indirect beneficial influence on reducing colon diseases including cancer [7]. The clear advantage presented by Australian green banana flour (GBF) includes a considerable total phenolic content (6 mg Gallic acid equivalent (GAE)/g) in Ladyfinger, (0.35 mg Quercetin equivalent (QE)/g) flavonoids in Ducasse, and a notable tannin content in Cavendish with 10 mg Catechin equivalent (CE)/g. Additionally, the complex carbohydrate profile shows high total starch (TS) (60.9%) and RS (45%) in Ladyfinger banana and DF content (39%) in the Ducasse variety [8].

A strong motivation for the current study was the annual waste of as much as 100,000 tons of green bananas due to inappropriate shapes and sizes that do not align with market and industry standards [9]. To reduce this waste, a combination of banana flour and other alternative flours has been suggested for use in the majority of starch-based food products, including pasta [10], bread [11], cake [12], and gluten-free products [13], in order to provide additional health benefits utilizing this functional ingredient. To date, limited research has examined both the pulp and peel of green bananas [14]. According to Bashmil et al. [8], the three Australian banana cultivars (Cavendish, Ladyfinger, and Ducasse) show significantly different (TS), RS, and DF content. As a result, each variety could uniquely impact the flour mixing behavior and final product quality. Therefore, studying the rheological characteristics of variant banana cultivars is a crucial step towards developing a high-quality functional bread.

As indicated by previous research on the effect of green banana inclusion in bread, the addition of 20% of green Ladyfinger (with high RS, 42.21%) improved bread volume, reduced hardness, improved sensory acceptance, and increased the slowly digestible starch fraction in crumbs. However, the banana addition caused darker bread color [15]. Furthermore, the 30% inclusion of green plantain flour from Costa Rica improved loaf volume and resulted in lighter bread crusts, softer crumb firmness, and regular porosity structure, and the optimization of water content, baking time, and temperature to maximize RS (3%), but increased darkness of the bread crumb [16]. Similarly, Bchir et al. [17] investigated the incorporation of 10% flesh fiber concentrate (FFC) from apple, pear, and date pomaces into wheat bread and its effects on dough performance and bread quality. Adding FFC significantly improved dough properties, including increased water absorption and stability, enhanced tenacity, and reduced extensibility and softening compared to the control dough. Bread made with FFC had varied crust and crumb colors, a comparable specific volume, and a more aerated crumb structure.

It is well established that the rheological properties of dough are crucial in the production of baked products since they influence the features and characteristics that are used to evaluate the quality of the bread-making process and the final bread products [18]. However, no studies have been conducted to evaluate the quality and nutritional value of bread fortified with different varieties of Australian green bananas. Therefore, this study aimed to examine the physicochemical properties of fortified bread including color values (L*, a*, and b*), crumb cell structure, TS, RS, and DF content. Additionally, the rheological behavior of wheat flour dough was investigated using the new Chopin MixoLab 2 device after the addition of varying amounts of Australian, green, Cavendish, Ladyfinger, and Ducasse banana flour substitutions (5, 10, 15, 25, and 30%).

## 2. Results and Discussion

### 2.1. MixoLab Analysis

**Figure 1:**
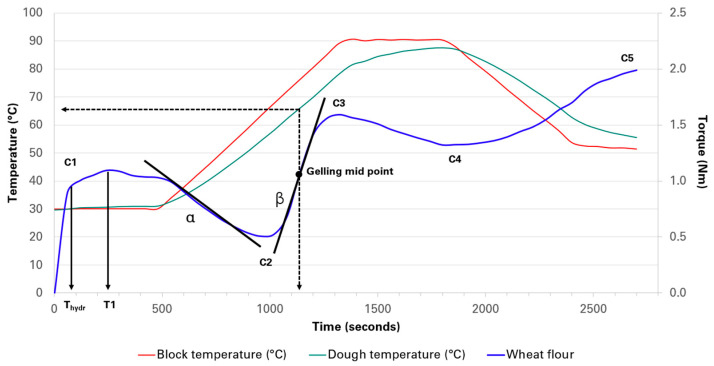
The MixoLab curve and the parameters analyzed from the standard sample (wheat flour). C1: water absorption; C2: protein weakening as a function of mechanical work and temperature; C3: starch gelatinization; C4: hot gel stability; C5: starch retrogradation in the cooling phase.

The Mixolab parameters including T_hydr_, T1, C1, water absorption, amplitude, stability, slope-α, C2, slope-β, C3, time to C3, gelling mid-point, C4, and C5 for GBF replacement levels (0–15%) are presented in Table 1. The 30–80 °C phase analysis monitored torque variations in mixtures of wheat flour, unripe banana flour, and water, as they were mixed and heated to create a gluten–starch complex. This analysis provides a valuable indicator of newly formed complexes between protein and starch, reliant on the characteristics of the protein polymers and the quantity and quality of amylose and amylopectin present [19]. The elevated temperature range of the MixoLab involved a notable variance among the flours derived from the combination of wheat and banana varieties. Specific banana cultivar flours were evaluated alongside our control flour (a plain commercial flour) to examine early mixing process events that may affect dough development and behavior, as illustrated in Figure 1. The Mixolab curves of samples presented noticeable differences as the temperature change occurred (curves of banana–wheat flour mixtures) as shown in the Supplementary Materials (Figures S1–S3). Additionally, the MixoLab parameters for 20, 25, and 30% substitution levels are displayed in Table S1 of the Supplementary Materials.

#### 2.1.1. Flour Water Absorption Capacity and Stability

The hydration of wheat proteins is essential for dough development. It is crucial for transforming wheat protein structures into a gluten network. In addition, starch and DF are recognized as having a critical influence on moisture content due to their superior water-binding capacity, which might impact the quality of the final product [20]. In the first phase of the MixoLab analysis, the water absorption percentage, initial time of hydration (T_hydr_), dough development time (T1), and stability were significantly affected by banana flour substitution up to 15% (Table 1 and Table S1 in Supplementary Materials for higher substitution levels, 20–30%).

In this study, results showed that increasing GBF substitution levels elevated flour water absorption. Compared to control bread, 10% and 15% Ladyfinger and 15% Ducasse had the highest water absorption with 63.9, 67.0, and 63.5%, respectively. The complex carbohydrates in unripe bananas influence dough water absorption mainly because of their elevated levels of RS and DF. RS and DF have a substantial concentration of hydroxyl groups, boosting their ability to bind water and promoting the formation of hydrogen bonds with water molecules. This improves the dough’s ability to hold water, leading to heightened water absorption. Enhanced water retention supports the dough’s elasticity, consistency, and fermentation rate, resulting in a softer and more pliable dough [21].

The greater RS content in the Australian Ladyfinger (48.9%) was demonstrated by Bashmil et al. [8] when compared to Cavendish and Ducasse. Their research, about RS and total dietary fiber (TDF) content in Australian green banana cultivars (Cavendish, Ladyfinger, and Ducasse), confirmed the effect of Ladyfinger addition on the flour water absorption and the considerable increase we found in this study. The Ducasse showed the highest TDF concentration (38.7%), which can be the reason for the substantial rise in the water absorption level with 15% substitution. Consequently, the optimal water absorption level for dough development to achieve the C1 torque (1.1 ± 0.00 Nm) varied based on the level of banana flour replacement and the cultivar variations.

These findings strongly agree with the Mohebbi et al. [22] study on the effects of RS and beta-glucan (BG) on the dough properties and bread-making characteristics. They observed the positive correlation between increasing BG and RS powder concentration and the high dough water absorption. Furthermore, investigating the effect of wheat bran dietary fiber (WBDF) on wheat flour dough rheological characteristics revealed a significant increase in water absorption (68.5%) at a 12% WBDF addition level [23].

The excessive water use at substitution levels up to 35% GBF resulted in higher moisture content in the bread, making it sticky and potentially promoting the rapid growth of microorganisms [16]. Therefore, we recommend using only a 10% GBF substitution. At this substitution level, no significant increase in water absorption was observed compared to the control bread. Additionally, the drying technique used for banana flour significantly affects its moisture content. Asif-Ul-Alam et al. [24] investigated the effects of hot air drying and freeze drying on the physicochemical and functional properties of green banana (*Musa sapientum*) flour and its incorporation into biscuits. Their findings indicated that hot air-dried banana flour had higher moisture content compared to freeze-dried flour, resulting in greater water-holding capacity for the hot air-dried flour. Considering these factors, using freeze-dried banana flour and limiting substitution levels to 10% can help minimize excessive water retention and reduce the potential for microbial activity in bread.

The T_hydr_ for wheat and banana flour in the current investigation demonstrated a significantly shorter T_hydr_ when adding banana flour. All banana varieties in different substitution levels had the same effect compared to control bread (3.6 min; see Table 1). However, dough development time (T1), the required time for the dough to reach the C1 torque, showed the opposite trend, in which 15% additions of all cultivars took longer development time (7.7, 6.5, and 6.1 min for Ladyfinger, Cavendish, and Ducasse, respectively, Table 1) than 5 and 10% additions. The high amount of DF and RS in GBF, with great water-binding capacities, quickly absorb and retain water when mixed into the dough, and hence lead to a rapid initial hydration phase [21].

In a mixture of wheat flour and unripe banana flour, the added banana flour competes with wheat flour for available water. Due to its higher affinity for water, unripe banana flour can be argued to hydrate rapidly, leaving less water available for gluten proteins in wheat flour. This faster initial absorption can create a shorter hydration time. Nevertheless, the RS and DF content lengthened dough development time, rendering it more cohesive and less extensible. Consequently, additional time and effort may be required to achieve the ideal consistency of the dough [25].

Interestingly, bread enrichment with 10 and 15% of all banana varieties considerably enhanced dough stability and resistance to overmixing, with 15% Ladyfinger showing the highest stability (7.31 min) compared to control bread (6.97 min). This high stability indicated that the wheat–banana flour was stronger than the wheat flour, even though the wheat flour substitution with GBF reduced the gluten content, which is generally considered responsible for maintaining the stability of the dough. Our results reported that the fortification with up to 15% Australian banana flour could improve the bread dough stability and did not weaken the gluten network as some researchers had anticipated [21,26,27,28]. However, no significant change was noticed in the dough elasticity (amplitude).

In contrast to our study, in previous research, a Farinograph indicated a decrease in dough stability when wheat flour was replaced by ripe American banana [28], unripe banana (*Musa padisiaca*) from Taiwan [21], pumpkin [26], and white grape pomace [27] in different quantities. This was attributed to the dilution of the gluten proteins when DF is introduced, which inhibits the proteins from forming a robust network [29]. Chikpah et al. [30] investigated the effect of the partial substitution of wheat flour with orange-fleshed sweet potato (OFSP) (10–50%) and pumpkin flour (10–40%) on bread dough rheological characteristics and found that stability declined with increasing proportions of OFSP and pumpkin flour. Other authors have attributed the increase in dough stability resulting from the addition of apple pomace to improved intermolecular interactions among DF, water, and gluten [31]. The extended stability of the composite dough is thus complex as the interaction between water and flour components, such as starch and fiber, can contribute to variable responses to mixing forces [26].

#### 2.1.2. Gluten Weakening

C2 torque identifies protein weakening, represented by the minimum torque value generated when the dough experiences concurrent elevations in temperature and mechanical stress. In this study, control dough (0% GBF) and 5% Cavendish, Ladyfinger, and Ducasse had the greatest C2 torque values (0.52, 0.49, 0.51, and 0.50 Nm) compared to the other fortification levels, contrary to the anticipated strengthening effect of GBF. In addition, slope-α (speed of protein weakening under the effect of heat + overmixing) did not change significantly with different substitution levels except the 15% Ladyfinger bread, which showed the greatest rate of gluten weakening (−0.055 Nm/s). This may be related to the dilution of gluten by GBF, resulting in dough degradation, the disruption of the starch–gluten network structure, and a reduction in resistance to overmixing [32]. Thus, the replacement of a portion of wheat flour with GBF led to a decrease in protein content, which was significantly greater at higher substitution levels up to 30% as presented in Table S1, Supplementary Materials.

**Table 1:** Dough rheological characteristics of green banana-enriched bread at different levels using MixoLab.

Samples	Substitution (%)	T_hydr_ (min)	T1 (min)	C1 (Nm)	Water Absorption (%)	Amplitude (Nm)	Stability (min)	Slope-α (Nm/min)
Control bread	0	3.63 ± 0.00 ^b^	7.08 ± 0.00 ^a^	1.10 ± 0.00 ^a^	62.8 ± 0.00 ^cd^	0.073 ± 0.00 ^a^	6.97 ± 0.00 ^cd^	−0.095 ± 0.00 ^b^
Cavendish	5	4.85 ± 0.15 ^a^	4.19 ± 0.02 ^b^	1.10 ± 0.03 ^a^	61.7 ± 0.00 ^e^	0.092 ± 0.04 ^a^	6.87 ± 0.15 ^d^	−0.100 ± 0.00 ^b^
Ladyfinger		1.13 ± 0.14 ^de^	5.91 ± 0.20 ^ab^	1.13 ± 0.03 ^a^	62.8 ± 0.00 ^cd^	0.101 ± 0.02 ^a^	7.04 ± 0.08 ^bd^	−0.099 ± 0.00 ^b^
Ducasse		1.11 ± 0.04 ^e^	5.28 ± 0.25 ^ab^	1.12 ± 0.03 ^a^	62.8 ± 0.52 ^d^	0.115 ± 0.03 ^a^	7.02 ± 0.04 ^cd^	−0.100 ± 0.00 ^b^
Cavendish	10	1.31 ± 0.04 ^ce^	5.66 ± 0.05 ^ab^	1.09 ± 0.01 ^a^	61.7 ± 0.00 ^e^	0.107 ± 0.01 ^a^	6.85 ± 0.02 ^d^	−0.111 ± 0.01 ^b^
Ladyfinger		1.36 ± 0.06 ^ce^	5.91 ± 1.51 ^ab^	1.08 ± 0.01 ^a^	63.9 ± 0.00 ^b^	0.105 ± 0.01 ^a^	7.29 ± 0.09 ^a^	−0.085 ± 0.00 ^b^
Ducasse		1.57 ± 0.42 ^cd^	5.50 ± 1.16 ^ab^	1.10 ± 0.03 ^a^	62.8 ± 0.58 ^d^	0.114 ± 0.03 ^a^	7.16 ± 0.06 ^a-c^	−0.102 ± 0.01 ^b^
Cavendish	15	1.36 ± 0.03 ^ce^	6.54 ± 0.99 ^ab^	1.05 ± 0.02 ^a^	61.7 ± 0.00 ^e^	0.094 ± 0.03 ^a^	7.23 ± 0.08 ^ab^	−0.105 ± 0.00 ^b^
Ladyfinger		1.70 ± 0.06 ^c^	7.68 ± 0.32 ^a^	0.94 ± 0.02 ^b^	67.0 ± 0.00 ^a^	0.109 ± 0.02 ^a^	7.31 ± 0.02 ^a^	−0.055 ± 0.00 ^a^
Ducasse		1.43 ± 0.05 ^ce^	6.14 ± 1.67 ^ab^	1.07 ± 0.08 ^a^	63.5 ± 0.00 ^bc^	0.110 ± 0.01 ^a^	7.26 ± 0.09 ^a^	−0.090 ± 0.02 ^b^
**Samples**	**Substitution (%)**	**C2 (Nm)**	**Slope-β** **(Nm/min)**	**C3 (Nm)**	**Time to C3 (min)**	**Gelling Mid-point (°C)**	**C4 (Nm)**	**C5 (Nm)**
Control bread	0	0.52 ± 0.00 ^a^	0.110 ± 0.00 ^d^	1.61 ± 0.00 ^c^	21.7 ± 0.00 ^c^	66.0 ± 0.00 ^d^	1.29 ± 0.00 ^cd^	1.89 ± 0.00 ^c^
Cavendish	5	0.49 ± 0.01 ^ac^	0.151 ± 0.02 ^cd^	1.71 ± 0.00 ^b^	22.5 ± 0.48 ^ab^	66.0 ± 0.01 ^d^	1.29 ± 0.00 ^cd^	1.95 ± 0.04 ^c^
Ladyfinger		0.51 ± 0.01 ^a^	0.155 ± 0.00 ^cd^	1.73 ± 0.00 ^b^	22.5 ± 0.04 ^ab^	68.0 ± 0.08 ^c^	1.37 ± 0.05 ^bc^	2.13 ± 0.09 ^b^
Ducasse		0.50 ± 0.00 ^ab^	0.147 ± 0.03 ^cd^	1.74 ± 0.00 ^b^	22.3 ± 0.15 ^b^	66.0 ± 0.01 ^d^	1.26 ± 0.03 ^d^	1.86 ± 0.00 ^c^
Cavendish	10	0.43 ± 0.01 ^d^	0.219 ± 0.00 ^b^	1.77 ± 0.01 ^ab^	22.9 ± 0.13 ^a^	69.2 ± 0.24 ^b^	1.38 ± 0.05 ^ab^	2.13 ± 0.08 ^b^
Ladyfinger		0.47 ± 0.00 ^bc^	0.174 ± 0.01 ^bc^	1.72 ± 0.00 ^b^	22.7 ± 0.18 ^ab^	68.1 ± 0.15 ^c^	1.41 ± 0.02 ^ab^	2.19 ± 0.04 ^ab^
Ducasse		0.46 ± 0.00 ^c^	0.184 ± 0.03 ^bc^	1.75 ± 0.02 ^b^	22.6 ± 0.12 ^ab^	68.0 ± 0.01 ^c^	1.23 ± 0.07 ^d^	1.85 ± 0.09 ^c^
Cavendish	15	0.43 ± 0.01 ^d^	0.325 ± 0.01 ^a^	1.84 ± 0.01 ^a^	22.8 ± 0.04 ^ab^	70.1 ± 0.09 ^a^	1.45 ± 0.01 ^a^	2.28 ± 0.05 ^a^
Ladyfinger		0.42 ± 0.02 ^d^	0.197 ± 0.00 ^bc^	1.70 ± 0.04 ^bc^	22.7 ± 0.05 ^ab^	68.1 ± 0.17 ^c^	1.45 ± 0.04 ^a^	2.22 ± 0.07 ^ab^
Ducasse		0.42 ± 0.03 ^d^	0.229 ± 0.01 ^b^	1.74 ± 0.05 ^b^	22.6 ± 0.06 ^ab^	69.2 ± 0.23 ^b^	1.24 ± 0.06 ^d^	1.96 ± 0.02 ^c^

Mean values in the same column followed by different letters are significantly different (*p* < 0.05). C1: water absorption; T_hydr_: initial time of hydration; T1: dough development time; C2: protein weakening as a function of mechanical work and temperature; C3: starch gelatinization; C4: hot gel stability; C5: starch retrogradation in the cooling phase. Nm: newton-meters.

Furthermore, the polyphenol content of unripe banana [4,8] could play a substantial role in gluten network structural changes. The complex interactions between proteins, carbohydrates, and phenolic compounds might significantly affect the functionality of bread components. Besides the interactions among phenolic acids that lead to oligomer formation, proteins are notably suitable interaction partners owing to their nucleophilic nature and the presence of either hydrophobic or hydrophilic regions [33]. The interactions between some proteins and phenolic acids influence their physicochemical qualities (e.g., protein solubility, hydrophobicity, isoelectric point) and techno-functional characteristics (e.g., emulsifying, foaming, gelling) [34]. Polyphenols can diminish disulfide cross-linking in gluten proteins, thus reducing the strength of the gluten network. This may result in a more pliable dough with less mixing duration [35].

#### 2.1.3. Starch Gelatinization and Gel Stability

The C3 stage accounts for starch gelatinization where the starch granules, when heated in the presence of water, absorb water, swell, and lose their crystalline structure, eventually leading to the dissolution of starch molecules into the surrounding water [36]. This process is crucial in cooking and baking since it helps set the structure of the bread crumb, influencing the bread’s softness and moisture retention, giving the bread a firm, crispy crust. This C3 stage features the gelling mid-point (Figure 1), an important parameter since it refers to the temperature or point at which 50% of the starch granules within a sample have gelatinized but have not yet reached full dissolution or breakdown. In bread making, the gelling mid-point indicates the optimal point where the crumb structure starts to stabilize, and the interactions between starch and gluten begin to set the final bread characteristics [36]. In this study, several factors including the type and amount of starch and its interaction with protein, DF, and polyphenols contributed to these features.

Our MixoLab analysis revealed that compared to the control bread, C3 torque increased significantly with the addition of GBF (as displayed in Table S1, Supplementary Materials). However, 10 and 15% Cavendish inclusions reached the greatest maximum torque during starch gelatinization. Similarly, the β slope (starch granule gelatinization speed) increased in the dough samples with 15% GBF, particularly Cavendish, which had the highest gelling mid-point temperature with 70.1 °C, while the 10% Cavendish and 15% Ducasse showed the second highest temperature (both 69.2 °C) (Table 1). Moreover, the total duration for starch to fully gelatinize (T3, Table 1) was increased substantially at all GBF addition levels compared to control bread. The positive effect of GBF inclusion extended to increasing the stability of the resulting gel (C4) in the 10 and 15% Cavendish and Ladyfinger additions (Table 1).

These findings agree with those of Pereira et al.’s [37] work, which indicated that the increased concentration of carbohydrates, including RS and fibers in GBF, facilitated the formation of a more structured and robust protein network than pure wheat flour. Consequently, the enhancement in the gelatinization of the GBF component could be linked to improved emulsion stability and water retention capacity. In other experiments involving mixtures of wheat flour and pectin-enriched material (PEM) derived from apple pomace, it was noted that PEM interacts with starch granules, resulting in increased starch pasting parameters with the incorporation of PEM [38]. Furthermore, PEM is considered to elevate the endothermic starch gelatinization peak, signifying that extra energy is necessary for breaking down starch granules. Additionally, the increase in starch gelatinization temperature is argued to be due to the high-water absorption of PEM, leading to reduced water availability for starch gelatinization.

Another important factor is the drying method used in GBF preparation. Investigating the properties of GBF (*Musa* spp. *ABB*) from Thailand processed via various drying methods such as air drying, freeze drying, and extrusion revealed that GBF derived from air and freeze drying exhibited superior RS content (69–72%), enhanced water-holding capacity, increased viscosity of starch paste, and elevated gelatinization temperatures (76–88 °C) compared to the extrusion method [39]. Moreover, the thermal characteristics of various starches differed based on environmental growth conditions, variety differences, and stages of ripening. Unripe green bananas from Tanzania, specifically Mzuzu (plantains) and Malindi (*Cavendish* sp.), exhibited broader gelatinization ranges compared to other types [40], suggesting more heterogeneity of their starch granules and potential differences in the organization of starch components inside the granules [41]. Variables of this type could explain the differences between Australian banana cultivars discovered in this project.

Flour gel stability (C4) is associated with the stability of the starch gel and is influenced by the natural α-amylase present in wheat flour. The combined effects of RS, DF, and polyphenols can contribute synergistically to inhibit α-amylase activity and enhance the stability of the starch gel in bread. This is particularly beneficial for bread texture attributes by maintaining crumb structure, enhancing softness, and improving moisture retention. RS does not break down easily into smaller glucose units. As a result, the presence of RS in bread decreases the substrate availability for α-amylase, leading to lower enzyme activity [42]. Additionally, RS contributes to a stronger and more stable gel network in the bread due to the structural integrity and crystalline regions of RS, which are not easily hydrolyzed by α-amylase [43]. Similarly, soluble and insoluble fibers can physically obstruct enzyme access to starch, leading to lower α-amylase activity. Moreover, certain soluble fibers might create a viscous environment that reduces the enzyme’s diffusion and activity rate [44].

The GBF is rich in polyphenols [4], which can bind to α-amylase, reducing its activity by blocking the enzyme’s active sites or altering its conformation. This leads to a slower rate of starch hydrolysis in bread, preserving the gel structure [45]. Also, polyphenols can form complexes with starch and gluten proteins, reinforcing the network and enhancing gel stability. This cross-linking effect reduces the susceptibility of starch to enzymatic breakdown [35].

#### 2.1.4. Starch Retrogradation

The C5 phase in Mixolab profiles is associated with the cooling period, where amylose and amylopectin begin to realign and form a more ordered crystalline structure. As the temperature drops, the gelatinized starch attempts to return to a semi-crystalline state [46]. In our study, bread enrichment with GBF significantly raised dough resistance during retrogradation, particularly 10 and 15% Cavendish and Ladyfinger. High torque during this phase suggests that the retrograded starch gel is stronger and more resistant to deformation (Table 1 and Table S1, Supplementary Materials). This generally correlates with higher crystallinity of the retrograded starch and a greater extent of the recrystallization of amylose molecules. Essentially, the dough or starch gel is becoming more rigid or solid-like as it cools [47].

The C5 torque value was strongly linked with the C4 torque value. The alterations in the C4 and C5 values may be influenced by changes in starch, including gelatinization and retrogradation. However, the structure and function of the gluten network are considered to be influenced primarily by temperature variations, with cooling following heating resulting in an enhancement of the solid-like characteristics (storage modulus) of gluten dough [48]. As a result, the two components (gluten and starch) may jointly influence the C4 and C5 characteristics of dough.

### 2.2. Resistant and Non-Resistant Starch and Dietary Fiber Content in GBF-Enriched Bread

White bread does not contain the nutrients found in the whole grain fraction and contains a large amount of quickly digestible starch due to the starch gelatinization that occurs as a result of baking the dough at temperatures above 70 °C while maintaining a high water content of about 65% [49]. Thus, the effect of white loaf bread enrichment with GBF (5, 10, and 15%) on RS, non-resistant starch (N-RS), TS, and TDF content was investigated in this study. This standard substitution level was chosen according to the dough rheological parameters studied earlier in this project.

As presented in Table 2, the control bread has significantly the lowest RS content (1.32%), which agrees with previous research [50,51,52]. As the amount of GBF increased in bread, the RS and TDF percentages increased substantially. Among all fortification percentages, replacing 15% of wheat flour with GBF resulted in a significant increase in TS, RS, and TDF proportions. The inclusion of 15% Ducasse and 15% Ladyfinger showed the greatest RS amount (2.58% and 2.56%, respectively), followed by 15% Cavendish (2.26%), while the highest TS was found in 15% Cavendish and Ladyfinger with 71.4% and 84.3%, respectively. On the other hand, the 5% Cavendish bread had the lowest RS% with 1.59% among other enriched bread samples. Whereas the 5% Ladyfinger showed the lowest TS and TDF amount (64.4% and 3.8%, respectively), the 15% Ladyfinger had the highest TS and TDF amount (84.3% and 9.1%, respectively) compared to other cultivars. The great TS amount in the 15% Ladyfinger bread could be a result of the thinner peel of Ladyfinger bananas compared to Cavendish and Ducasse bananas; thus, the flour generated from Ladyfinger slices had a slightly greater pulp/skin ratio that contributed to the increased TS content reported in this study.

Increasing the TS content in banana bread, particularly through the addition of GBF, can enhance its nutritional value and functionality. Starch provides a steady energy source and contributes to satiety, making it a valuable macronutrient in food products. Compared to previous studies, our data demonstrate that the TS content of all banana cultivars at the 15% substitution level examined in this study was markedly higher than that of Nigerian plantain bananas, as shown by Eke-Ejiofor and Kiin-Kabari [53]. With a 20% substitution of GBF, the TS content in their bread was 58.20%, which is lower than that reported for Australian bananas, as mentioned in Table 2. Additionally, a study by Khoozani et al. [11] on bread prepared with 20% replacement with whole green banana (Cavendish, *Musa AAA* group) from New Zealand revealed a TS content of 53.0%. This highlights the variability in TS content across different banana cultivars and products, emphasizing the importance of cultivar selection in functional food development.

DF is an essential component of a healthy diet, offering numerous benefits, including improved digestion, enhanced gut health, and reduced risk of chronic diseases such as cardiovascular disease and type 2 diabetes. In this study, fortifying bread with GBF increased TDF in all cultivars. However, the 20% substitution with whole green banana (Cavendish of *Musa AAA* group) from New Zealand resulted in significantly lower TDF content (3.8%) [11] compared to our 15% Cavendish (8.0%), Ladyfinger (9.1%), and Ducasse (7.1%). Besides the impact of banana varieties on TDF content, DF can interact with other components like gluten, starch, or fats, affecting fiber retention and its availability in the final bread product [54].

Roman et al. [55] found that the RS concentration in bread produced with 20% banana starch cannot exceed 2% *w*/*w*. It was discovered that substituting 25% of wheat flour with unripe banana pulp powder resulted in a maximum 3.22% of RS in the bread, as reported in [56]. The GBF that was used in bread by Juarez-Garcia et al. [52] had an additional 6% gluten added. The study found that the bread’s RS was just 6.7%, which was explained by the wheat’s low level of RS (only 17%). As discovered by Agama-Acevedo et al. [57], only 2.5% RS of the examined bread crust was still available.

Several factors can affect the RS content in bread. For example, proteins from wheat (glutenin, gliadin) have the potential to generate disulphide bonds that result in a continuous coating surrounding starch granules following boiling, and slow the process of starch digestion [58]. According to Berti et al. [59], gluten-containing breads had lower postprandial glucose levels than gluten-free loaves because of the protein network encasing the starch.

After the bread samples have been cooled and the amylo-lipid linkages have formed, the retrogradation process results in RS types 3 and 5, respectively [60]. Baking causes starch molecules to become disaggregated, but cooling causes them to reassociate, producing structures reinforced by hydrogen bonding that are more resistant to digestion. This process is known as gelatinization [61]. In vitro and in vivo studies have shown that retrograded amylose is resistant to digestion by α-amylase [62]. Additionally, the inclusion complexes formed by amylose and fats have been demonstrated to be more resistant to digestion than the individual components themselves [63]. Some starches (particularly those with a high amylose content) naturally contain amylose–lipid complexes [64]. It is also possible to create them using hydrothermal treatments such as baking, in the presence of exogenous or endogenous lipids including monoglycerides, fatty acids, and surfactant lipids [63].

Kneading, proofing, and baking are all part of the bread-making process. The most common method of incorporating RS substances into the mix is to replace starchy material with RS materials. Only a few studies have investigated the RS content in the final product. The type and quantity of RS produced by baking can be significantly altered, so this step is crucial. RS2 from green bananas is not heat-stable and will not withstand baking; hence, a considerable portion of RS will be lost during baking [55,65]. Furthermore, RS2 in bananas diminishes with ripening due to the endogenous α-amylase’s conversion of RS2 into reducing sugar [65]. It has been observed that α-amylase is most active between 8 and 38 °C, and that it begins to denature at 38 °C and is completely denatured after 5 min at 100 °C [66]. Thus, drying will be important to stabilize RS2 in banana flour and preserve it therein. The effect of oven and freeze drying on banana flour DF content was studied by Pico et al. [67], who found that oven drying results in an insoluble DF concentration of 26.8%, whereas freeze drying results in a much higher insoluble fiber content of 43.3%. Even though banana RS is lost during baking, banana starch has been found to have a molecular composition that can produce slowly digested starch in bread crumbs after baking retrogradation, some of which could reach the colon as RS3 [55].

Apart from the composition of RS ingredients, how bread is processed can have an impact on the level of RS generation. Bread that was baked at a low temperature and for an extended period of time had much more RS than bread baked at a higher temperature and for a shorter time [16,51]. RS has also been found to increase when more water is added to the bread mix. Increased starch retrogradation (RS3) occurs after cooling of gelatinized starch when the water content in the dough is higher [16]. It has been found that RS3 in wheat bread is larger at refrigerator temperatures than at ambient or frozen temperatures [68]; thus, for some starches, refrigeration temperatures may improve their RS properties in breads.

### 2.3. Flour and Bread Physical Properties

#### 2.3.1. Flour and Bread Color

**Figure 2:**
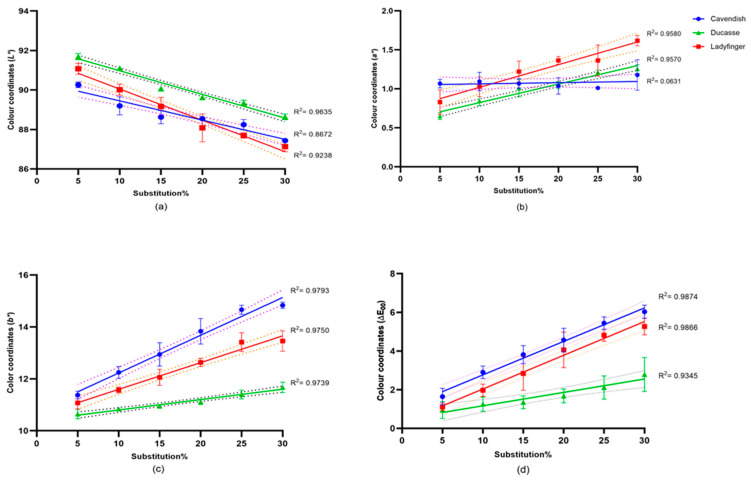
Correlation coefficients of green banana flour (GBF) substitution (0, 5, 10, 20, 25, 30%) and the L*, a*, and b* color space and ΔE_00_ measured by a Nix Spectro L sensor. (**a**): L*; (**b**): a*; (**c**): b*; (**d**): ΔE_00_. The dashed lines represent the standard error (SE) of the measurements.

**Figure 3:**
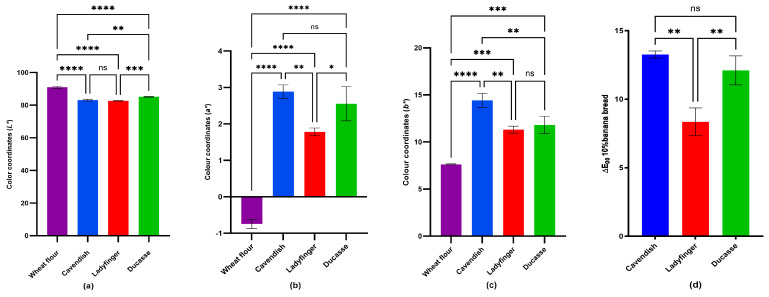
L*, a*, and b* color space and ΔE_00_ of 10% banana bread measured by Nix Spectro L sensor. (**a**): L*; (**b**): a*; (**c**): b*; (**d**): ΔE_00_; ****: *p* < 0.0001 (extremely significant); ***: *p* < 0.001 (very highly significant); **: *p* < 0.01 (highly significant); *: *p* < 0.05 (significant); and ns: non-significant.

The acceptability of bread in the marketplace and among consumers is significantly influenced by its color, which is primarily determined by its raw ingredients, additions, fermentation, and baking conditions. The results of wheat flour color tests with GBF inclusions (0, 5, 10, 15, 20, 25, 30%) and the 10% GBF-enriched bread are shown in Figure 2 and Figure 3. The 10% GBF-enriched standard was chosen based on the moderate effects observed in the MixoLab analysis. The results in Figure 2 showed a strong positive correlation between the GBF substitution level and flour color. The L* values decreased significantly with the increase in GBF content. Especially, the blends of 30% Cavendish and Ladyfinger additions had the darkest color, while 0% blends had the lighter color.

Thus, we recommend using a small substitution level of 10% of GBF, which has been shown in our study to have only a moderate effect on bread color while still providing nutritional benefits. This substitution level strikes a balance between maintaining the visual appeal of the bread and enhancing its functional properties, such as increased RS and DF content.

Furthermore, with the increase in GBF content, the greenness (a*) and the yellowness (b*) of the flour increased significantly with 30% Ladyfinger showing the greatest a* value (1.6), while the 30% Cavendish had the highest b* value (14.8). These differences could be related to the banana cultivar variations. In the total color difference (ΔE) method, which accurately reflects human perception for color differences [69], all Cavendish and Ladyfinger inclusion levels and 30% Ducasse additions showed noticeable differences (ranged from 2 to 10), in which the higher the value, the more obvious the difference for naked eyes [70].

In Figure 3, our findings demonstrated the color difference in 10% GBF bread of the three banana cultivars. The L* results revealed a highly significant reduction (*p* < 0.0001) compared to the control (wheat bread), particularly the Cavendish and Ladyfinger (83.1 and 82.7, respectively), which means that the flour substitution with 10% GBF resulted in a notably darker bread. Measuring the greenness (a*), even though all varieties showed a highly significant increase (*p* < 0.0001), Cavendish and Ducasse had a greater increase in a* values than Ladyfinger (2.9, 2.6, and 1.8, respectively). Similarly, the yellowness of samples (b*) was substantially higher in 10% banana bread than in the control bread, with Cavendish and Ducasse showing a greater increase (14.4 and 11.8, respectively). Generally, the ΔE indicated that, again, Cavendish and Ducasse addition caused a ΔE value greater than 10, which indicates that the colors are distinctly different and easily distinguishable by the human eye [70]. Ladyfinger still exhibited a noticeable difference (8.4).

Our results were consistent with those of Zhang et al. [71]. In their study, various proportions of Agaricus bisporus powder (ABP) (0, 2, 4, 6, and 8%) were incorporated with wheat flour to produce bread, and bread quality including bread color tests (L*, a*, and b*) were investigated. Their findings indicated that the L* value diminished markedly with increased ABP content, while the a* and b* values increased. Moreover, research by Khoozani et al. [11] revealed the same L* result reduction after the use of New Zealand whole green banana flour (WGBF) (Cavendish of *Musa AAA*) in bread at fortification levels of 10%, 20%, and 30%. However, they found the opposite trend in the a* and b* results, in which the inclusion of 10% WGBF caused a significant decrease in a* and b* values compared with the control bread. This could be attributed to the effect of cultivar variation.

The darkness of the GBF bread may be ascribed to several factors. The inclusion of various types and concentrations of polyphenols in dough influences the color and texture of baked goods differently. According to Ou et al. [72], food byproducts, for example, fruit pomace, abundant with polyphenols and DF, are increasingly utilized in baked goods as sources of antioxidants and functional components. While these plant components could boost the antioxidant activity of wheat bread, nonetheless, the majority of these compounds could negatively affect the sensory attributes, particularly product color. Polyphenol oxidation and the interaction of their oxidized derivatives with amino acids may influence color development. Heat, metals, polyphenol oxidase, or high pH facilitates the oxidation of polyphenols into quinones. Quinones can interact with amino acids and proteins via the Maillard process, leading to the production of brown polymeric colors [72].

The greater carbohydrate content in GBF may have contributed to Maillard reaction acceleration, which initiates with the interaction of reducing sugar and free amino groups or peptide/protein-bound amino side chains. However, caramelization is the carbohydrate reaction that does not involve amino compounds. Both reactions induce favorable and unfavorable alterations in the final product [73]. This explained the darker color of the 10% Cavendish bread in this project, since Cavendish had the greatest amount of N-RS (23.4%) compared to Ladyfinger and Ducasse [8], and this type of starch can interact with amino acids during baking and result in a darker bread.

#### 2.3.2. Crumb Cell Structure Analysis

**Figure 4:**
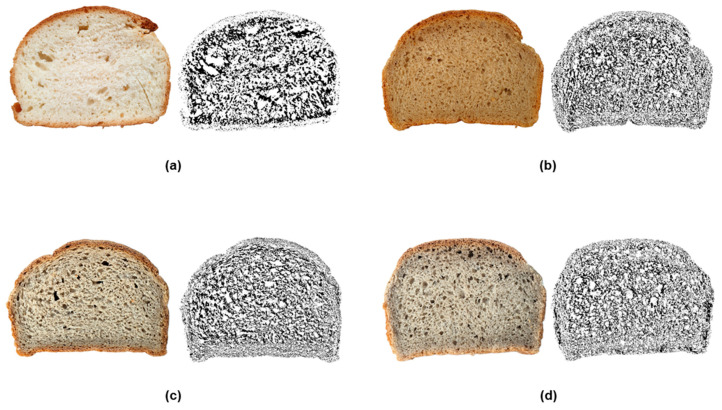
Cell structure analysis of 10% banana bread using MATLAB R2024a bread texture analyzer. (**a**): wheat flour bread; (**b**): Cavendish bread; (**c**): Ladyfinger bread; (**d**): Ducasse bread. Black-and-white photos: air pocket segmentation.

**Figure 5:**
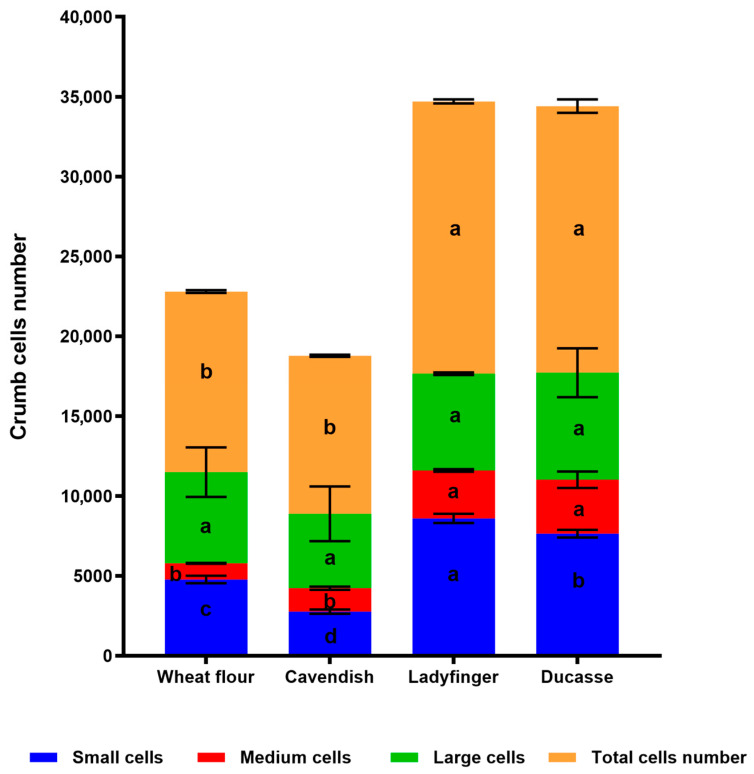
A stacked graph showing the mean values of the number of air spaces assessed from images of wheat and 10% banana bread (Figure 4). Different letters ^a–d^ describe significant differences between samples based on the ANOVA and Tukey’s honest significant difference (HSD) post hoc test (*p* < 0.05).

**Figure 6:**
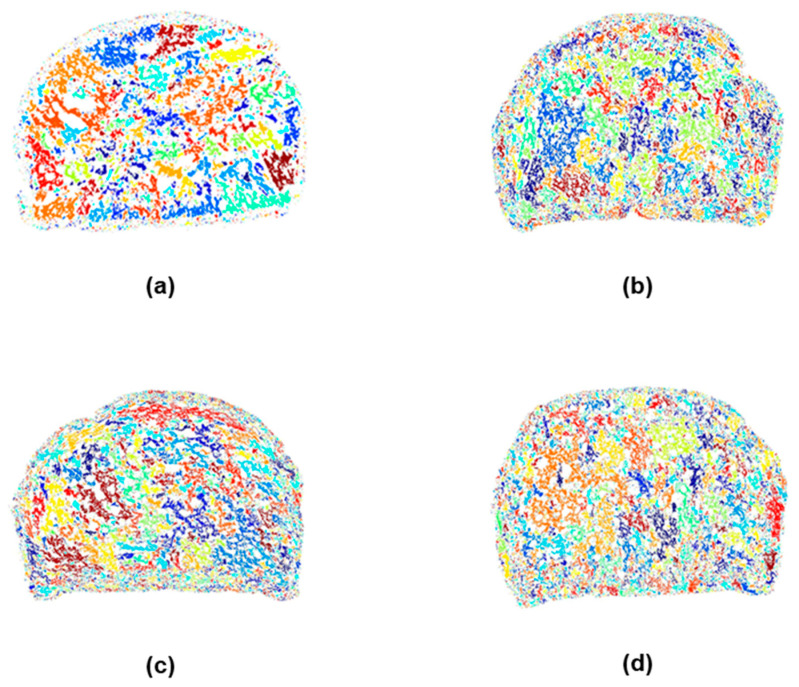
Cell size segmentation and color coding of 10% banana bread using MATLAB R2024a bread texture analyzer. (**a**): wheat flour bread; (**b**): Cavendish bread; (**c**): Ladyfinger bread; (**d**): Ducasse bread.

The bread texture analyzer based on a MATLAB R2024a application was used to identify connected regions in a digital image of a bread sample and to calculate density description parameters [74]. MATLAB used statistical image segmentation to generate the images in Figure 4, Figure 5 and Figure 6, which show the distribution and size of crumb air spaces in 10% GBF bread compared to control wheat bread. Figure 5 illustrates substantial variation (*p* < 0.0001) across samples regarding the small, medium, and total air space counts assessed by computer vision techniques. Ladyfinger and Ducasse bread exhibited the highest medium and total quantity of air holes, whereas the control and Cavendish samples demonstrated the lowest. Likewise, Ladyfinger bread exhibited the greatest quantity of small air pockets, whilst Cavendish displayed the least. These physical properties can directly influence the texture of GBF-enriched bread, and consequently, consumer satisfaction and sensory evaluations.

Furthermore, Figure 4 and Figure 6 reveal cell-size segmentation and color coding of 10% banana bread. Bread cell segmentation is a critical step in analyzing the crumb structure of bread, providing insights into texture, pore distribution, and overall quality. Black-and-white photos (Figure 4) simplify segmentation by focusing solely on intensity (brightness) differences, making them effective for uniform bread crumb structures where contrast between air pores and the crumb matrix is high.

However, color coding images (Figure 6) provide additional chromatic information, which can display a convenient fingerprint of the bread crumb and pore spaces by leveraging differences in hue, saturation, and brightness [75]. Consequently, the color map in Figure 6 demonstrates the unique alveoli distribution differences between various banana cultivar breads because they contained a different range and quantity of region sizes, and regions of the same size might not have shared the same color on different images.

In terms of crumb cell size and distribution, the incorporation of GBF into bread formulations generally results in a reduction in crumb cell size and a more uniform distribution. This effect can be attributed to the dough’s increased viscosity and decreased gas-holding capacity, which are influenced by the high DF and RS content. The higher dough stiffness limits the expansion of gas bubbles during proofing and baking, ultimately producing smaller air pockets within the crumb structure [76,77].

Moreover, at low substitution levels, <20%, the addition of GBF to bread formulations can result in a slight increase in the number of crumb cells. This is attributed to the ability of DF to act as nucleation sites for gas bubble formation during proofing. On the other hand, at higher substitution levels, the total number of crumb cells and bread volume may decrease, likely due to reduced gas retention and increased dough rigidity, which hinder the expansion and stabilization of gas bubbles [22,78].

Additionally, the high water-binding capacity of GBF could affect the number of bread cells by modifying dough hydration, gluten network formation, and gas retention properties. At the 10% substitution levels, the high water-binding capacity enhances dough hydration, creating a cohesive matrix that stabilizes gas bubbles during proofing, potentially increasing the number of crumb cells, as shown in Ladyfinger and Ducasse. However, it is expected that at higher substitution levels, excessive water binding reduces the availability of free water, weakening gluten development and gas retention. This leads to a reduced expansion and stabilization of gas bubbles, resulting in fewer and irregular crumb cells in the final bread structure [79].

## 3. Materials and Methods

### 3.1. Green Banana Flour Production

The banana powder was produced from Australian-grown green bananas (Cavendish, Ladyfinger, and Ducasse), incorporating both banana pulp and skin, purchased from several local markets in Melbourne. The bananas used in this study were classified as Stage 1 on the Commercial Standard Color Chart for banana maturation, which is defined as “all green” [80]. To effectively manage banana sap during the slicing process, we thoroughly washed the green bananas with distilled water before slicing to eliminate surface latex, then sliced the whole banana to a thickness of 2 mm and quickly immersed the slices in a 0.5% (*w*/*v*) citric acid solution to inhibit oxidation and discoloration and subsequently drained it. Furthermore, slicing was carried out efficiently to reduce sap release, and equipment was regularly cleaned to prevent latex accumulation, thus ensuring high-quality slices for flour manufacture. Citric acid was used to mitigate enzymatic browning by decreasing the pH, thus establishing an environment that is less favorable to the polyphenol oxidase (PPO) activity, the enzyme accountable for browning reactions. By reducing PPO activity, citric acid could minimize the oxidation of phenolic compounds into quinones, which subsequently polymerize to produce brown colors. This treatment assists in maintaining the natural color and quality of banana slices throughout processing. Following four hours in a −80 °C freezer, banana samples were transferred to a freeze dryer (Zirbus VaCo5 System, Bad Grund, Germany) at −50 °C and a pressure of 0.5 hPa for 72 h. The dried green banana slices were further ground with a coffee grinder (Breville Smart Grinder TM Pro, model BCG820BSSXL, Melbourne, VIC, Australia) to produce banana flour in an average particle size of 200 µm, which was then stored in plastic containers and maintained at 4 °C until assessment [8].

### 3.2. Banana Bread Preparation

The bread was prepared in the food processing laboratory at the Faculty of Science, School of Agriculture, Food and Ecosystem Sciences, University of Melbourne, Australia, using the Basic Straight-Dough Bread-Baking Method (AACC Method 10-09.01) [81]. The formula comprised 100% flour (with or without substitutes), 63–76% water, 2% salt, 2% yeast, 4% sugar, and 3% fat, with all percentages calculated depending on the weight of the flour. The ingredients were subsequently combined using a stand mixer (Kenwood Titanium Chef Baker XL Stand Mixer White KVL65001WH, Havant, UK) at low speed for 3 min, then at medium speed for 10 min, and eventually at high speed for an additional 10 min until a smooth, elastic dough was achieved. Afterwards, the dough underwent bulk fermentation for 60 min at 27–30 °C until it doubled in size, and was subsequently degassed, formed, and proofed for 60 min at 30–35 °C. The baking was conducted at 170 °C for a duration of 60 min. Finally, the bread was allowed to cool on a rack at room temperature (21–25 °C) for 1 h prior to slicing or packaging. Banana peel and pulp flour were used in the recipe with different replacement levels of 5%, 10%, and 15% (*w*/*w*). A control bread was prepared using 100% wheat flour. The banana flour was used during the dough mixing process.

### 3.3. Rheological Tests of Wheat Flour and GBF

The rheological behavior of doughs was obtained using different substitution levels of GBF (0, 5, 10, 15, 20, 25, and 30%), analyzed using the standard option “Chopin+” protocol on the Mixolab, with the following settings: a kneading speed of 80 rpm, mixer temperature of 30 °C, heating rate of 2 °C/min, and total analysis time of 45 min. The parameters obtained from the recorded curves involve time of hydration (T_hydr_, min), dough development time (T1, min), dough maximum consistency during development (C1, Nm), water absorption to reach C1 (%), dough elasticity (amplitude, Nm), dough stability (min), speed of protein weakening under the effect of heat + overmixing (slope-α, Nm/min), protein weakening (C2, Nm), starch gelatinization speed (slope-β, Nm/min), starch gelatinization (C3, Nm), gelling mid-point (°C), time to reach C3 (T3, min), amylolytic activity and gel stability (C4, Nm), and starch retrogradation (C5, Nm). The water absorption (%) of flour mixtures followed the standard determination from Mixolab. The analysis was performed three times for each sample. The parameters obtained for each sample were compared with the control sample (wheat flour).

### 3.4. RS and N-RS Analysis

The standardized procedure employed to quantify RS was AACC 32-40.01/AOAC 2002.02. The process was conducted using a Megazyme Kit (Megazyme International Ireland Ltd., Bray, Ireland). In this technique, materials (100 mg) were combined with a sodium maleate buffer (pH 6.0) containing pancreatic α-amylase and amyloglucosidase (AMG). Subsequently, samples were incubated in a shaking water bath at 37 °C for 4 h, during which N-RS was hydrolyzed to d-glucose by the activity of the two enzymes. The reaction was terminated by the addition of ethanol (99% *v*/*v*). The mixture was centrifuged for 10 min at 3000 rpm with two rinses with aqueous ethanol (50% *v*/*v*). The supernatant was removed, and the pellet (RS) was dissolved in 2 M KOH in an ice/water bath by vigorous stirring for 20 min. The solution was neutralized with a sodium acetate buffer, and the RS was hydrolyzed to glucose using AMG in a water bath for 30 min at 50 °C. After sample centrifugation, an aliquot was combined with a glucose oxidase/peroxidase reagent (GOPOD) and incubated at 50 °C for 20 min. Ultimately, the absorbance of each solution was compared to that of the reference standard (blank solution) at 510 nm. The N-RS was quantified by mixing all supernatant solutions acquired post-initial incubation and adjusting the volume to 100 mL with 100 mM sodium acetate buffer (pH 4.5). Aliquots of these solutions were then incubated with the AMG solution for 20 min at 50 °C. After the inclusion of the GOPOD reagent, all sample solutions were incubated for an additional 20 min, and then the concentration of N-RS was determined by measuring the absorbance at 510 nm. The TS content comprises both RS and N-RS [82].

### 3.5. Total Dietary Fiber Content (TDF)

TDF was evaluated following the AOAC method 985.29 (AOAC 1990). Samples were gelatinized using a heat-stable α-amylase (pH 6.0, 95 °C, 30 min) and thereafter subjected to sequential enzymatic digestion with protease (pH 7.5, 60 °C, 30 min) and amyloglucosidase (pH 4.5, 60 °C, 30 min) to eliminate protein and starch. TDF was precipitated using ethanol, followed by washing with ethanol and acetone, and eventually dried before weighing the residue. Results were adjusted for protein and ash content [83].

### 3.6. Color Test

The color values (L*, a*, b*) of wheat/banana flour (0, 5, 10, 15, 20, 25, and 30%) and 10% GBF (Cavendish, Ladyfinger, and Ducasse banana) bread crumbs were measured using a Nix Spectro L color sensor (Nix Sensor Ltd., Hamilton, ON, Canada). Three color readings were taken for each measurement group for each banana cultivar, and their average values were reported. The total color difference (ΔE) was calculated using the formula

$$\Delta E=\sqrt{\left\{{\left(\Delta L*\right)}^{2}+{\left(\Delta a*\right)}^{2}+{\left(\Delta b*\right)}^{2}\right\}}$$

This metric quantifies the overall perceptual color difference between the samples and a reference, based on lightness (L*), redness-greenness (a*), and yellowness-blueness (b*) values. The ΔE results were classified as follows: values between 0 and 1 indicate color differences imperceptible to the human eye, 1 < ΔE < 2 represent slight differences visible to trained observers, 2 < ΔE < 3.5 indicate noticeable differences even for unexperienced observers, 3.5 < ΔE < 5 signify substantial differences, and more than 5 show that colors are distinctly different [69,84].

### 3.7. Digital Image Texture Analysis

Images of three bread slices of each treatment were taken using the 3 cameras of a Samsung Galaxy S20+ (Samsung, Seoul, Republic of Korea). Slices were placed in an area with controlled lighting that provided consistency, and minimal shadows and reflections. The slices were positioned on a flat, white, non-glossy surface to ensure that the texture was clearly visible. The background of the image, namely all pixels that were not part of the slice, was removed using Adobe Photoshop rel.21.0.2 (Adobe Inc., San Jose, CA, USA). The processed images were saved in jpg format. To assess the texture, we developed a MATLAB texture analysis application, the bread texture analyzer (BTA) [74], using MATLAB software R2024a (MathWorks Inc., Natick, MA, USA). The software used digital images of the control and enriched bread cross-section and could run the analysis in a single- or multiple-file mode. It deployed a statistical image segmentation method to divide image pixels into foreground and background regions, followed by a post-process region analysis to assess the texture of samples. Additionally, the software applied the colourmap technique to visually distinguish the connected regions by area size within the image. A colourmap in MATLAB R2024a is a matrix of colors used to map data values to colors in visualizations.

### 3.8. Statistical Analysis

The results of this study were presented as the means ± standard deviation (SD) of three independent assessments utilizing three replicates. A one-way analysis of variance (ANOVA) and Tukey’s test were performed to ascertain statistically significant difference between the control and the three green banana cultivar breads with respect to the dough rheological behavior, TDF, RS, N-RS, TS, bread color evaluations, and crumb texture analysis (*p* ≤ 0.05). Minitab 19 (Minitab^®^ for Windows Release 19, Minitab Inc., Chicago, IL, USA) and GraphPad Prism 10 (Prism 10.3.1, GraphPad Software Inc., San Diego, CA, USA) were used for the data analysis and graph formations. Furthermore, for the MATLAB R2024a texture analysis application, the bread texture analyzer (BTA) was used to deploy a statistical image segmentation method and assess bread texture. All presented findings were corrected with blank or control values.

## 4. Conclusions

This study emphasizes the potential of GBF as a functional component in bread formulations, providing improved nutritional and physicochemical features while preserving satisfactory dough processing characteristics. Significant differences in the GBF attributes of the different banana cultivars were evident. The inclusion of GBF, especially from Ladyfinger and Cavendish cultivars, markedly enhanced water absorption, dough stability, and RS levels. Moreover, GBF additions also affected bread color and crumb structure, due to the elevated DF and RS levels in GBF, which altered dough rheology and product quality. Overall, our findings endorse the feasibility of using GBF in wheat-based bread to enhance DF and RS consumption, consistent with nutritional improvement objectives as well as contributing to food waste reduction. Further research should concentrate on assessing customer acceptability and examining the effects of storage conditions to improve the practical implementation of GBF in commercial bread manufacturing.

## Figures and Tables

**Table 2:** Starch and dietary fiber content of green banana-enriched bread.

Samples	Substitution (%)	N-RS (g/100 g)	RS (g/100 g)	TS (g/100 g)	TDF (g/100 g)
Control bread	0	64.0 ± 0.14 ^f^	1.32 ± 0.01 ^f^	65.3 ± 0.14 ^g^	3.8 ± 0.39 ^g^
Cavendish	5	62.9 ± 0.06 ^gh^	1.59 ± 0.01 ^e^	64.5 ± 0.07 ^h^	5.9 ± 0.24 ^f^
Ladyfinger	5	62.6 ± 0.07 ^h^	1.76 ± 0.01 ^d^	64.4 ± 0.08 ^h^	3.8 ± 0.38 ^g^
Ducasse	5	63.1 ± 0.07 ^g^	1.90 ± 0.01 ^c^	65.0 ± 0.07 ^g^	6.4 ± 0.08 ^d^
Cavendish	10	72.0 ± 0.18 ^b^	1.89 ± 0.01 ^c^	73.9 ± 0.18 ^b^	5.9 ± 0.18 ^e^
Ladyfinger	10	70.8 ± 0.24 ^c^	1.90 ± 0.01 ^c^	72.7 ± 0.24 ^c^	7.1 ± 0.11 ^c^
Ducasse	10	68.9 ± 0.07 ^d^	1.89 ± 0.01 ^c^	70.8 ± 0.07 ^e^	5.9 ± 0.28 ^ef^
Cavendish	15	69.1 ± 0.13 ^d^	2.26 ± 0.01 ^b^	71.4 ± 0.14 ^d^	8.0 ± 0.39 ^b^
Ladyfinger	15	81.7 ± 0.13 ^a^	2.56 ± 0.02 ^a^	84.3 ± 0.15 ^a^	9.1 ± 0.45 ^a^
Ducasse	15	65.1 ± 0.24 ^e^	2.58 ± 0.01 ^a^	67.7 ± 0.25 ^f^	7.1 ± 0.42 ^c^

Mean values in the same column followed by different letters are significantly different (*p* < 0.05). RS: resistant starch; N-RS: non-resistant starch; TS: total starch; TDF: total dietary fiber; 5%, 10%, and 15%, substitution levels of freeze-dried green banana flour with wheat flour.

## Data Availability

The data presented in this study are available in this manuscript and Supplementary Materials.

## Supplementary Materials

The following supporting information can be downloaded at: https://www.mdpi.com/article/10.3390/plants14020207/s1, Figure S1: MixoLab analysis for Cavendish–wheat flour mixtures; Figure S2: MixoLab analysis for Ladyfinger–wheat flour mixtures; Figure S3: MixoLab analysis for Ducasse–wheat flour mixtures; Table S1: Dough rheological characteristics of green banana-enriched bread at different levels using MixoLab.
