# Supplementary material for: The Physicochemical and Rheological Properties of Green Banana Flour–Wheat Flour Bread Substitutions
Source: Plants (Basel). 2025 Jan 13;14(2):207. doi: 10.3390/plants14020207 (PMC11768194; doi:10.3390/plants14020207)
Supplement: Supplementary file 1 [file plants-14-00207-s001.zip › plants-3397358-supplementary.pdf]

plants

Article

The Physicochemical and Rheological Properties of Green Banana Flour-Wheat Flour Bread Substitutions

Yasmeen M. Bashmil [1,2], Frank Bekes [3], Michael Ruderman [2], Hafiz A. R. Suleria [2], Rudi Appels [2,*], and Frank R. Dunshea [2,4]

[1] Faculty of Human Sciences and Design, Department of Food and Nutrition, King Abdulaziz University, Jeddah 21589, Saudi Arabia
[2] Faculty of Science, School of Agriculture, Food and Ecosystem Sciences, The University of Melbourne, Parkville, VIC 3010, Australia
[3] FBFD Pty Ltd., Sydney, NSW 2151, Australia
[4] Faculty of Biological Sciences, University of Leeds, Leeds LS2 9JT, UK
* Correspondence: rudi.appels@unimelb.edu.au; Tel.: +61-404-448-337

Supplementary Materials

(a) MixoLab analysis for Cavendish-wheat flour mixtures

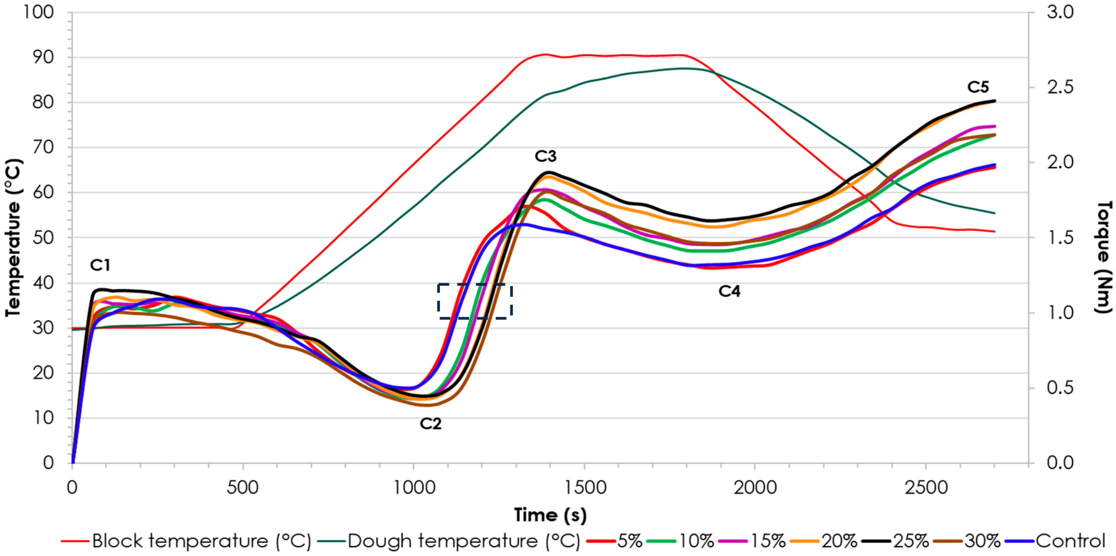

Figure S1. MixoLab curve and the analyzed parameters from Cavendish-wheat flour mixtures at 5, 10, 15, 20, 25, and 30% fortification levels. C1: water absorption; C2: protein weakening as a function of mechanical work and temperature; C3: starch gelatinization; C4: hot gel stability; C5: starch retrogradation in the cooling phase. The boxed region of the MixoLab traces indicates a region that characterizes the midpoints of the starch gelling process that is diagnostic of the changes induced by the GBF substitutions.

(b) MixoLab analysis for Ladyfinger-wheat flour mixtures

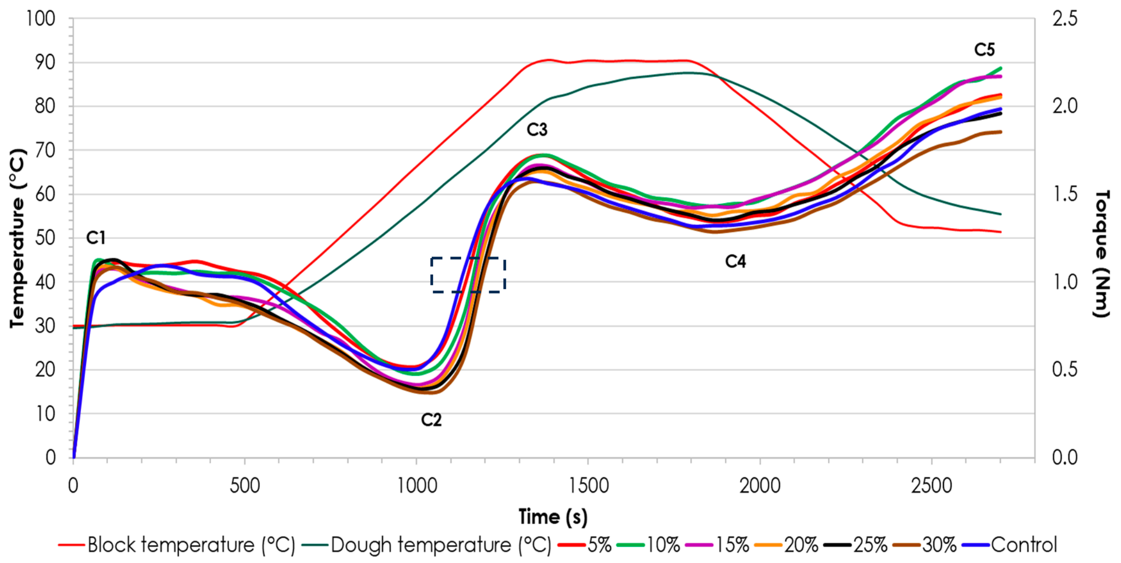

Figure S2. MixoLab curve and the analyzed parameters from Ladyfinger-wheat flour mixtures at (5, 10, 15, 20, 25, and 30%) fortification levels. C1: water absorption; C2: protein weakening as a function of mechanical work and temperature; C3: starch gelatinization; C4: hot gel stability; C5: starch retrogradation in the cooling phase. The boxed region of the MixoLab traces indicates a region that characterizes the midpoints of the starch gelling process that is diagnostic of the changes induced by the GBF substitutions.

(c) MixoLab analysis for Ducasse-wheat flour mixtures

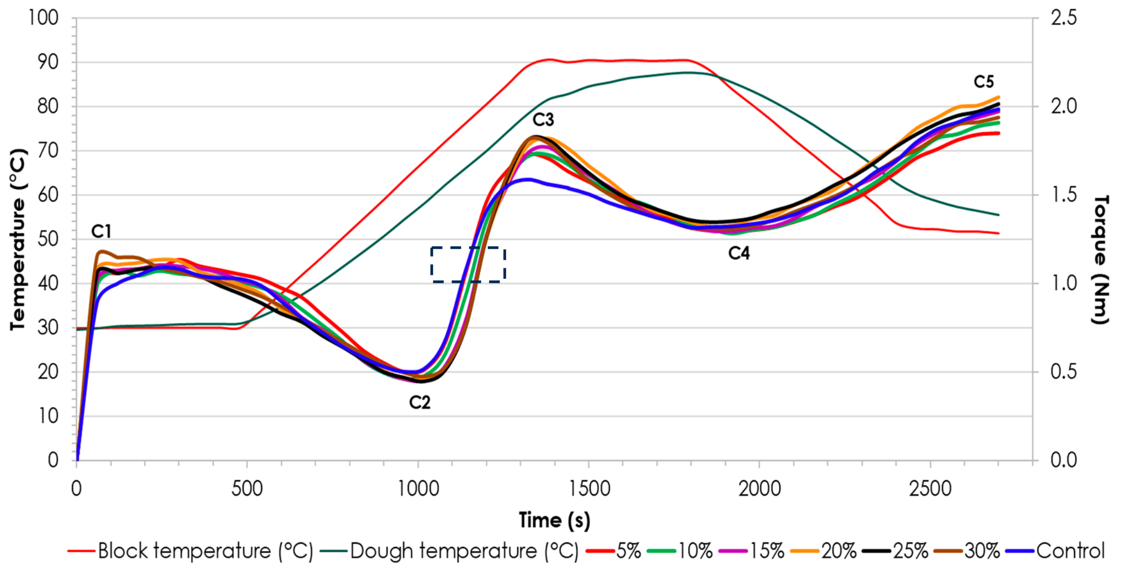

Figure S3. MixoLab curve and the analyzed parameters from Ducasse-wheat flour mixtures at (5, 10, 15, 20, 25, and 30%) fortification levels. C1: water absorption; C2: protein weakening as a function of mechanical work and temperature; C3: starch gelatinization; C4: hot gel stability; C5: starch retrogradation in the cooling phase. The boxed region of the MixoLab traces indicates a region that characterizes the midpoints of the starch gelling process that is diagnostic of the changes induced by the GBF substitutions.

Table S1. Dough rheological characteristics of green banana enriched bread at different levels using MixoLab.

Samples	Substitution (%)	T_{hydr} (min)	T1 (min)	C1 (Nm)	Water absorption (%)	Amplitude (Nm)	Stability (min)	Slope-α (Nm/min)
Control bread	0	3.63 ± 0.00 ^{ab}	7.08 ± 0.00 ^{ab}	1.10 ± 0.00 ^{ab}	62.8 ± 0.00 ^{fg}	0.073 ± 0.00 ^{b}	6.97 ± 0.00 ^{bd}	-0.095 ± 0.00 ^{bf}
Cavendish		4.85 ± 0.15 ^{a}	4.19 ± 0.02 ^{b}	1.10 ± 0.03 ^{ab}	61.7 ± 0.00 ^{g}	0.092 ± 0.04 ^{b}	6.87 ± 0.15 ^{cd}	-0.100 ± 0.00 ^{cf}
Ladyfinger	5	1.13 ± 0.14 ^{e}	5.91 ± 0.20 ^{ab}	1.13 ± 0.03 ^{a}	62.8 ± 0.00 ^{fg}	0.101 ± 0.02 ^{ab}	7.04 ± 0.08 $^{a-c}$	-0.099 ± 0.00 ^{cf}
Ducasse		1.11 ± 0.04 ^{e}	5.28 ± 0.25 ^{ab}	1.12 ± 0.03 ^{a}	62.8 ± 0.52 ^{g}	0.115 ± 0.03 ^{ab}	7.02 ± 0.04 $^{a-c}$	-0.100 ± 0.00 ^{cf}
Cavendish		1.31 ± 0.04 ^{e}	5.66 ± 0.05 ^{ab}	1.09 ± 0.01 ^{ab}	61.7 ± 0.00 ^{g}	0.107 ± 0.01 ^{ab}	6.85 ± 0.02 ^{cd}	-0.111 ± 0.01 ^{f}
Ladyfinger	10	1.36 ± 0.06 ^{de}	5.91 ± 1.51 ^{ab}	1.08 ± 0.01 ^{ab}	63.9 ± 0.00 ^{fg}	0.105 ± 0.01 ^{ab}	7.29 ± 0.09 ^{ab}	-0.085 ± 0.00 ^{af}
Ducasse		1.57 ± 0.42 ^{de}	5.50 ± 1.16 ^{ab}	1.10 ± 0.03 ^{ab}	62.8 ± 0.58 ^{g}	0.114 ± 0.03 ^{ab}	7.16 ± 0.06 $^{a-c}$	-0.102 ± 0.01 ^{df}
Cavendish		1.36 ± 0.03 ^{e}	6.54 ± 0.99 ^{ab}	1.05 ± 0.02 $^{a-c}$	61.7 ± 0.00 ^{g}	0.094 ± 0.03 ^{ab}	7.23 ± 0.08 ^{ab}	-0.105 ± 0.00 ^{ef}
Ladyfinger	15	1.70 ± 0.06 ^{ce}	7.68 ± 0.32 ^{a}	0.94 ± 0.02 ^{de}	67.0 ± 0.00 ^{de}	0.109 ± 0.02 ^{ab}	7.31 ± 0.02 ^{ab}	-0.055 ± 0.00 ^{a}
Ducasse		1.43 ± 0.05 $^{a-c}$	6.14 ± 1.67 ^{ab}	1.07 ± 0.08 ^{ab}	63.5 ± 0.00 ^{fg}	0.110 ± 0.01 ^{ab}	7.26 ± 0.09 ^{ab}	-0.090 ± 0.02 ^{bf}
Cavendish		1.95 ± 0.05 ^{be}	4.29 ± 0.02 ^{b}	1.09 ± 0.00 ^{ab}	61.7 ± 0.00 ^{g}	0.150 ± 0.00 ^{a}	6.65 ± 0.00 ^{d}	-0.096 ± 0.00 ^{bf}
Ladyfinger	20	1.48 ± 0.04 ^{de}	6.54 ± 1.93 ^{ab}	0.91 ± 0.03 ^{e}	70.0 ± 0.00 ^{bc}	0.111 ± 0.02 ^{ab}	7.30 ± 0.13 ^{ab}	-0.068 ± 0.00 ^{ab}
Ducasse		3.17 ± 1.26 $^{a-d}$	6.10 ± 1.63 ^{ab}	1.05 ± 0.07 $^{a-d}$	65.4 ± 1.63 ^{ef}	0.117 ± 0.02 ^{ab}	7.25 ± 0.24 ^{ab}	-0.080 ± 0.01 $^{a-e}$
Cavendish		2.10 ± 0.50 ^{be}	4.25 ± 0.08 ^{b}	1.11 ± 0.01 ^{a}	62.3 ± 0.52 ^{g}	0.143 ± 0.02 ^{a}	6.63 ± 0.15 ^{d}	-0.097 ± 0.00 ^{bf}
Ladyfinger	25	1.61 ± 0.17 ^{de}	5.88 ± 1.40 ^{ab}	0.95 ± 0.03 ^{ce}	72.6 ± 0.00 ^{b}	0.126 ± 0.04 ^{ab}	7.29 ± 0.03 ^{ab}	-0.074 ± 0.00 $^{a-d}$
Ducasse		2.39 ± 1.19 ^{be}	6.97 ± 0.14 ^{ab}	1.03 ± 0.10 $^{a-d}$	67.9 ± 1.96 ^{ce}	0.125 ± 0.02 ^{ab}	7.38 ± 0.01 ^{a}	-0.079 ± 0.02 $^{a-e}$
Cavendish		1.49 ± 0.01 ^{de}	4.31 ± 0.10 ^{b}	0.99 ± 0.00 ^{be}	65.3 ± 0.58 ^{ef}	0.128 ± 0.00 ^{ab}	7.07 ± 0.00 $^{a-c}$	-0.089 ± 0.00 ^{bf}
Ladyfinger	30	1.65 ± 0.21 ^{ce}	4.29 ± 0.10 ^{b}	0.99 ± 0.01 ^{be}	76.1 ± 0.00 ^{a}	0.130 ± 0.01 ^{ab}	6.99 ± 0.34 ^{bd}	-0.076 ± 0.00 $^{a-e}$
Ducasse		1.36 ± 0.21 ^{de}	5.92 ± 1.52 ^{ab}	1.06 ± 0.01 ^{ab}	69.1 ± 2.71 ^{cd}	0.114 ± 0.01 ^{ab}	7.36 ± 0.02 ^{a}	-0.071 ± 0.01 $^{a-c}$

Mean values in the same column followed by different letters are significantly different ($p < 0.05$). C1: water absorption; T_{hydr}: initial time of hydration; T1: dough development time; (Nm): newton-meters.

Samples	Substitution (%)	C2 (Nm)	Slope-β (Nm/min)	C3 (Nm)	Time to C3 (min)	Gelling mid-point (° C)	C4 (Nm)	C5 (Nm)
Control bread	0	0.52 ± 0.00 ^{a}	0.110 ± 0.00 ^{j}	1.61 ± 0.00 ^{fg}	21.7 ± 0.00 ^{e}	66.0 ± 0.00 ^{f}	1.29 ± 0.00 ^{dg}	1.89 ± 0.00 ^{gh}
Cavendish		0.49 ± 0.01 $^{a-c}$	0.151 ± 0.02 ^{hi}	1.71 ± 0.00 ^{dg}	22.5 ± 0.48 ^{cd}	66.0 ± 0.01 ^{f}	1.29 ± 0.00 ^{dg}	1.95 ± 0.04 ^{fh}
Ladyfinger	5	0.51 ± 0.01 ^{ab}	0.155 ± 0.00 ^{i}	1.73 ± 0.00 ^{cg}	22.5 ± 0.04 ^{cd}	68.0 ± 0.08 ^{e}	1.37 ± 0.05 ^{cg}	2.13 ± 0.09 $^{b-e}$
Ducasse		0.50 ± 0.00 $^{a-c}$	0.147 ± 0.03 ^{i}	1.74 ± 0.00 $^{c-f}$	22.3 ± 0.15 ^{d}	66.0 ± 0.01 ^{f}	1.26 ± 0.03 ^{eg}	1.86 ± 0.00 ^{h}
Cavendish		0.43 ± 0.01 ^{dg}	0.219 ± 0.00 ^{ef}	1.77 ± 0.01 $^{b-e}$	22.9 ± 0.13 ^{ad}	69.2 ± 0.24 ^{d}	1.38 ± 0.05 $^{c-e}$	2.13 ± 0.08 $^{b-f}$
Ladyfinger	10	0.47 ± 0.00 ^{bd}	0.174 ± 0.01 ^{gi}	1.72 ± 0.00 ^{cg}	22.7 ± 0.18 ^{ad}	68.1 ± 0.15 ^{e}	1.41 ± 0.02 ^{cd}	2.19 ± 0.04 $^{b-d}$
Ducasse		0.46 ± 0.00 $^{c-e}$	0.184 ± 0.03 $^{f-h}$	1.75 ± 0.02 $^{c-f}$	22.6 ± 0.12 ^{bd}	68.0 ± 0.01 ^{e}	1.23 ± 0.07 ^{g}	1.85 ± 0.09 ^{h}
Cavendish		0.43 ± 0.01 $^{e-h}$	0.325 ± 0.01 ^{b}	1.84 ± 0.01 $^{a-d}$	22.8 ± 0.04 ^{ad}	70.1 ± 0.09 ^{c}	1.45 ± 0.01 ^{bc}	2.28 ± 0.05 ^{ab}
Ladyfinger	15	0.42 ± 0.02 $^{e-h}$	0.197 ± 0.00 ^{fg}	1.70 ± 0.04 ^{dg}	22.7 ± 0.05 ^{ad}	68.1 ± 0.17 ^{e}	1.45 ± 0.04 ^{bc}	2.22 ± 0.07 ^{ac}
Ducasse		0.42 ± 0.03 $^{e-h}$	0.229 ± 0.01 ^{df}	1.74 ± 0.05 $^{c-f}$	22.6 ± 0.06 ^{ad}	69.2 ± 0.23 ^{d}	1.24 ± 0.06 ^{fg}	1.96 ± 0.02 ^{ef}
Cavendish		0.43 ± 0.00 $^{e-h}$	0.427 ± 0.00 ^{a}	1.90 ± 0.00 ^{ab}	22.95 ± 0.06 $^{a-c}$	72.1 ± 0.08 ^{b}	1.57 ± 0.00 ^{ab}	2.41 ± 0.01 ^{a}
Ladyfinger	20	0.39 ± 0.01 ^{gi}	0.209 ± 0.00 ^{fg}	1.65 ± 0.03 ^{eg}	22.61 ± 0.35 ^{ad}	68.0 ± 0.06 ^{e}	1.41 ± 0.03 ^{cd}	2.12 ± 0.06 $^{b-f}$
Ducasse		0.41 ± 0.04 $^{f-h}$	0.259 ± 0.01 ^{de}	1.75 ± 0.11 $^{c-f}$	22.78 ± 0.02 ^{ad}	70.0 ± 0.01 ^{c}	1.27 ± 0.08 ^{eg}	1.99 ± 0.18 ^{eh}
Cavendish		0.44 ± 0.00 $^{d-f}$	0.435 ± 0.01 ^{a}	1.92 ± 0.01 ^{a}	23.15 ± 0.06 ^{a}	72.0 ± 0.01 ^{b}	1.60 ± 0.01 ^{a}	2.41 ± 0.01 ^{a}
Ladyfinger	25	0.39 ± 0.00 ^{hi}	0.266 ± 0.02 ^{cd}	1.65 ± 0.00 ^{eg}	22.86 ± 0.33 ^{ad}	69.0 ± 0.01 ^{d}	1.38 ± 0.02 $^{c-f}$	2.04 ± 0.07 ^{ch}
Ducasse		0.41 ± 0.03 $^{f-h}$	0.301 ± 0.01 ^{bc}	1.80 ± 0.06 $^{a-d}$	22.36 ± 0.11 ^{d}	69.0 ± 0.06 ^{d}	1.34 ± 0.00 ^{cg}	2.02 ± 0.02 ^{dh}
Cavendish		0.39 ± 0.00 ^{hi}	0.427 ± 0.00 ^{a}	1.81 ± 0.00 $^{a-d}$	23.11 ± 0.02 ^{ab}	73.0 ± 0.01 ^{a}	1.44 ± 0.00 ^{c}	2.20 ± 0.01 $^{b-d}$
Ladyfinger	30	0.37 ± 0.00 ^{i}	0.261 ± 0.01 ^{d}	1.60 ± 0.01 ^{g}	22.61 ± 0.11 ^{ad}	69.0 ± 0.06 ^{d}	1.30 ± 0.02 ^{dg}	1.90 ± 0.04 ^{gh}
Ducasse		0.46 ± 0.01 $^{c-e}$	0.303 ± 0.01 ^{bc}	1.86 ± 0.03 ^{ac}	22.42 ± 0.16 ^{cd}	69.0 ± 0.06 ^{d}	1.44 ± 0.12 ^{bc}	2.07 ± 0.12 $^{c-g}$

Mean values in the same column followed by different letters are significantly different ($p < 0.05$). C2: protein weakening as a function of mechanical work and temperature; C3: starch gelatinization; C4: hot gel stability; C5: starch retrogradation in the cooling phase. (Nm): newton-meters.
